# Genetic scores to stratify risk of developing multiple islet autoantibodies and type 1 diabetes: A prospective study in children

**DOI:** 10.1371/journal.pmed.1002548

**Published:** 2018-04-03

**Authors:** Ezio Bonifacio, Andreas Beyerlein, Markus Hippich, Christiane Winkler, Kendra Vehik, Michael N. Weedon, Michael Laimighofer, Andrew T. Hattersley, Jan Krumsiek, Brigitte I. Frohnert, Andrea K. Steck, William A. Hagopian, Jeffrey P. Krischer, Åke Lernmark, Marian J. Rewers, Jin-Xiong She, Jorma Toppari, Beena Akolkar, Richard A. Oram, Stephen S. Rich, Anette-G. Ziegler, for the TEDDY Study Group

**Affiliations:** 1 DFG–Center for Regenerative Therapies Dresden, Faculty of Medicine, Technische Universität Dresden, Dresden, Germany; 2 Institute of Diabetes Research, Helmholtz Zentrum München, Munich, Germany; 3 Forschergruppe Diabetes, Technical University of Munich, Klinikum Rechts der Isar, Munich, Germany; 4 Forschergruppe Diabetes e.V. at Helmholtz Zentrum München, Munich, Germany; 5 Health Informatics Institute, Morsani College of Medicine, University of South Florida, Tampa, Florida, United States of America; 6 Institute of Biomedical and Clinical Science, University of Exeter Medical School, Exeter, United Kingdom; 7 Institute of Computational Biology, Helmholtz Zentrum München, Munich, Germany; 8 Barbara Davis Center for Childhood Diabetes, University of Colorado Denver, Aurora, Colorado, United States of America; 9 Pacific Northwest Diabetes Research Institute, Seattle, Washington, United States of America; 10 Department of Clinical Sciences, Clinical Research Centre, Skåne University Hospital, Lund University, Malmo, Sweden; 11 Center for Biotechnology and Genomic Medicine, Medical College of Georgia, Augusta University, Augusta, Georgia, United States of America; 12 Department of Pediatrics, Turku University Hospital, Turku, Finland; 13 Department of Physiology, University of Turku, Turku, Finland; 14 National Institute of Diabetes and Digestive and Kidney Diseases, National Institutes of Health, Bethesda, Maryland, United States of America; 15 Clinical Islet Transplant Program, University of Alberta, Edmonton, Alberta, Canada; 16 National Institute for Health Research, Exeter Clinical Research Facility, Exeter, United Kingdom; 17 Center for Public Health Genomics, University of Virginia, Charlottesville, Virginia, United States of America; Chinese University of Hong Kong, CHINA

## Abstract

**Background:**

Around 0.3% of newborns will develop autoimmunity to pancreatic beta cells in childhood and subsequently develop type 1 diabetes before adulthood. Primary prevention of type 1 diabetes will require early intervention in genetically at-risk infants. The objective of this study was to determine to what extent genetic scores (two previous genetic scores and a merged genetic score) can improve the prediction of type 1 diabetes.

**Methods and findings:**

The Environmental Determinants of Diabetes in the Young (TEDDY) study followed genetically at-risk children at 3- to 6-monthly intervals from birth for the development of islet autoantibodies and type 1 diabetes. Infants were enrolled between 1 September 2004 and 28 February 2010 and monitored until 31 May 2016. The risk (positive predictive value) for developing multiple islet autoantibodies (pre-symptomatic type 1 diabetes) and type 1 diabetes was determined in 4,543 children who had no first-degree relatives with type 1 diabetes and either a heterozygous HLA DR3 and DR4-DQ8 risk genotype or a homozygous DR4-DQ8 genotype, and in 3,498 of these children in whom genetic scores were calculated from 41 single nucleotide polymorphisms. In the children with the HLA risk genotypes, risk for developing multiple islet autoantibodies was 5.8% (95% CI 5.0%–6.6%) by age 6 years, and risk for diabetes by age 10 years was 3.7% (95% CI 3.0%–4.4%). Risk for developing multiple islet autoantibodies was 11.0% (95% CI 8.7%–13.3%) in children with a merged genetic score of >14.4 (upper quartile; *n =* 907) compared to 4.1% (95% CI 3.3%–4.9%, *P <* 0.001) in children with a genetic score of ≤14.4 (*n =* 2,591). Risk for developing diabetes by age 10 years was 7.6% (95% CI 5.3%–9.9%) in children with a merged score of >14.4 compared with 2.7% (95% CI 1.9%–3.6%) in children with a score of ≤14.4 (*P <* 0.001). Of 173 children with multiple islet autoantibodies by age 6 years and 107 children with diabetes by age 10 years, 82 (sensitivity, 47.4%; 95% CI 40.1%–54.8%) and 52 (sensitivity, 48.6%, 95% CI 39.3%–60.0%), respectively, had a score >14.4. Scores were higher in European versus US children (*P =* 0.003). In children with a merged score of >14.4, risk for multiple islet autoantibodies was similar and consistently >10% in Europe and in the US; risk was greater in males than in females (*P =* 0.01). Limitations of the study include that the genetic scores were originally developed from case–control studies of clinical diabetes in individuals of mainly European decent. It is, therefore, possible that it may not be suitable to all populations.

**Conclusions:**

A type 1 diabetes genetic score identified infants without family history of type 1 diabetes who had a greater than 10% risk for pre-symptomatic type 1 diabetes, and a nearly 2-fold higher risk than children identified by high-risk HLA genotypes alone. This finding extends the possibilities for enrolling children into type 1 diabetes primary prevention trials.

## Abstract 2

Anette-Gabriele Ziegler and colleagues report their novel genetic risk score for identifying infants with a high risk of developing type 1 diabetes.

## Author summary

**Why was this study done?:**

- Prevention of childhood diseases such as type 1 diabetes is of medical importance.
- Prevention of type 1 diabetes might be best achieved by intervention prior to the development of islet autoantibodies, which define a pre-symptomatic disease stage.
- Early intervention requires tools such as measures of genetic risk that identify future cases.
- Risk for type 1 diabetes in the absence of a family history is currently identified by HLA genotyping, with maximum identified risk reaching around 5%.
- Genetic scores derived from multiple risk loci may improve risk stratification for pre-symptomatic type 1 diabetes.

**What did the researchers do and find?:**

- Two previously proposed genetic scores for type 1 diabetes risk were calculated for over 3,000 children without a family history of type 1 diabetes but with 1 of the 2 highest-risk HLA genotypes (heterozygous DR3 and DR4-DQ8 or homozygous DR4-DQ8) participating in the TEDDY cohort study, which prospectively follows children from birth for the development of islet autoantibodies and diabetes.
- We found that both of the genetic scores, and a merged genetic score that combined the features of both, stratified the risk for islet autoantibodies and diabetes in the children.
- The upper quartile of the merged genetic score was associated with a >10% risk for the pre-symptomatic stage of multiple islet autoantibodies, and almost half the children who developed pre-symptomatic or symptomatic diabetes were identified by this score.

**What do these findings mean?:**

- Combining genetic information from multiple risk loci can improve the prediction of diseases such as type 1 diabetes.
- A genetic risk score model is proposed that could be used to recruit infants into early type 1 diabetes primary prevention trials.
- The model provides a new paradigm for genetic screening and selection of at-risk infants that, together with family history and HLA genotyping, could identify up to 25% of future childhood cases of type 1 diabetes from less than 1% of newborns.

## Introduction

Precision medicine typically relies on our ability to identify individuals with precise genetic elements that define a disease. These elements may be used not only to select optimal treatment modalities, but also to identify individuals who may benefit from preventative interventions. In pediatric disease, current studies seeking to elucidate disease etiology, as well as clinical trials aimed at prevention, rely on identifying and enrolling infants with increased risk [1–7]. The risk for diseases such as allergy, type 1 diabetes, and celiac disease is often assessed in terms of family history [1–3,7], which, at best, identifies 10% of children who subsequently develop the condition [7,8].

In type 1 diabetes, genotypes in the human leukocyte antigen (HLA) DRB1, DQA1, and DQB1 loci are sometimes used to identify at-risk infants from the general population [2,9,10]. Risk is 5% in children with the 2 highest-risk HLA genotypes (DR3 and DR4-DQ8 or homozygous for DR4-DQ8), and 40% of cases of childhood type 1 diabetes have 1 of these 2 genotypes [11]. Although the HLA loci are the strongest genetic risk markers for type 1 diabetes, many other regions of the genome also confer susceptibility to type 1 diabetes [12]. Therefore, it is conceivable that risk stratification could be improved if risk is calculated according to genetic information derived from multiple genetic susceptibility regions [13,14].

We previously applied logistic regression to the Type 1 Diabetes Genetics Consortium (T1DGC) case–control dataset and developed a weighted genetic score derived from HLA and 40 type 1 diabetes susceptibility loci (Winkler score) [15]. Independently, a genetic score derived from HLA plus 25 susceptibility loci was developed in the UK using Wellcome Trust Case Control Consortium (WTCCC) data (Oram score) [16]. These studies suggested that the scores might improve our ability to predict and diagnose type 1 diabetes. Hence, genetic scores could become a new paradigm for stratifying type 1 diabetes risk and for recruitment into primary prevention trials, and provide a proof of principle for other diseases with multiple known genetic susceptibility markers. With this in mind, the 2 consortia joined efforts to determine how the 2 genetic scores and a merged score performed in a prospective study.

The Environmental Determinants of Diabetes in the Young (TEDDY) study, a multicenter cohort study set in Germany, Finland, Sweden, and the US, has intensively followed several thousand HLA-selected children from birth for the development of islet autoantibodies and of diabetes [17]. The presence of 2 or more islet autoantibodies (multiple islet autoantibodies) in genetically at-risk children defines a pre-symptomatic stage of type 1 diabetes where progression to type 1 diabetes is around 80% over 10 years [18,19]. TEDDY offers the unique opportunity to test the multiple-locus genetic scores in a prospectively studied cohort of children who have high-risk HLA genotypes in the absence of family history of type 1 diabetes [2,17]. The objective of our analysis was to determine whether the genetic scores could identify infants from the general population who had at least a 10% risk for type 1 diabetes, a risk threshold that has been used for primary prevention trials and that has previously only been achievable in infants with a family history of type 1 diabetes [3].

## Methods

### Case–control cohort

We reasoned that our target risk of 10% could only be achieved by applying our multi-locus genetic scores in individuals who had the highest-risk HLA genotypes. We obtained data for controls from the UK Biobank (https://www.ukbiobank.ac.uk/) [20] and data for controls and cases from the WTCCC [21], and calculated the Winkler and Oram scores in 4,371 non-diabetic individuals who were heterozygous for HLA DR3-DQA1*0501-DQB1*0201 and DR4-DQA1*030X-DQB1*0302 (HLA DR3/DR4-DQ8) or who were homozygous for HLA DR4-DQA1*030X-DQB1*0302 (HLA DR4-DQ8/DR4-DQ8) (controls) and 781 patients with type 1 diabetes who had 1 of these 2 genotypes (cases). UK Biobank participants were aged 40 to 69 years, and the WTCCC patients were all aged <50 years when sampled.

### TEDDY cohort

**Fig 1:**
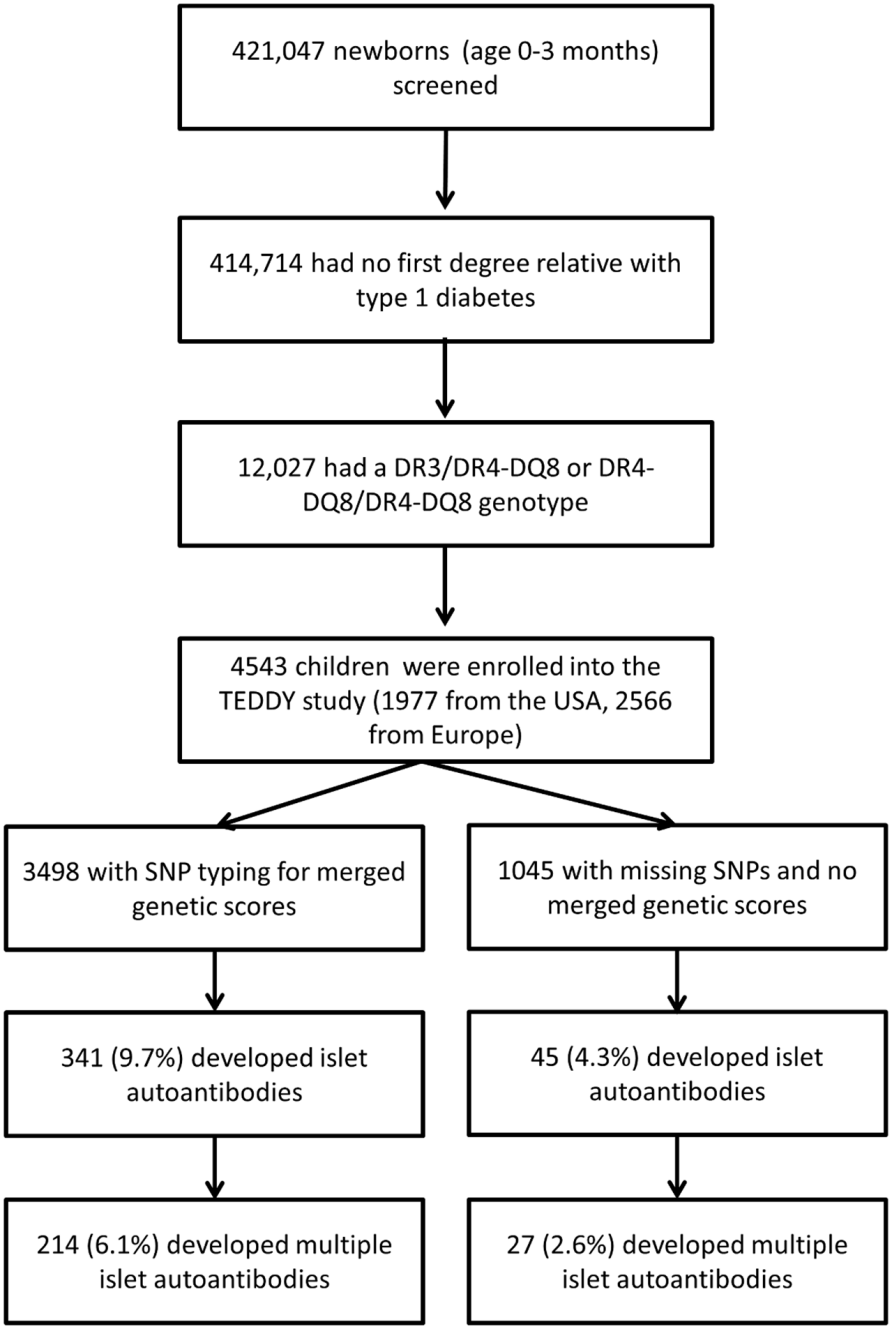
Flow diagram of the TEDDY study participants included in this analysis.

TEDDY is a prospective cohort study conducted at 3 centers in the US (Colorado, Georgia/Florida, and Washington) and 3 centers in Europe (Finland, Germany, and Sweden) [2,17]. Between 1 September 2004 and 28 February 2010, a total of 421,047 newborn children were screened for high-risk HLA genotypes for type 1 diabetes [22]. HLA genotype screening was conducted as previously described [22]. The families of children with type 1 diabetes risk HLA genotypes were invited to participate in the follow-up study in which blood samples were obtained every 3 months for the first 4 years and biannually thereafter for the analysis of islet autoantibodies (glutamic acid decarboxylase antibody [GADA], insulinoma antigen-2 antibody [IA-2A], and insulin autoantibodies [IAAs]). The HLA genotypes were confirmed by the central HLA Reference Laboratory at Roche Molecular Systems (Oakland, CA) for enrolled participants. The present report includes TEDDY children with the HLA DR3/DR4-DQ8 or the HLA DR4-DQ8/DR4-DQ8 genotype, without a first-degree relative with type 1 diabetes, if at least 1 blood sample was obtained after birth (Fig 1). This included 4,543 participants (2,278 [50.1%] girls). At analysis (follow-up to 31 May 2016), the median age of these children was 6.7 years (interquartile range, 2.5 to 8.6 years). Written informed consent was obtained for all study participants from a parent or primary caretaker for genetic screening and to participate in the prospective follow-up. The study was approved by local institutional review boards and is monitored by an external advisory board established by the US National Institutes of Health.

### TEDDY study outcomes

Islet autoantibodies (IAAs, GADA, and IA-2A) were measured by radiobinding assays every 3 months for the first 4 years and biannually thereafter. In the US, autoantibodies were measured at the Barbara Davis Center for Childhood Diabetes at the University of Colorado Denver reference laboratory. In Europe, autoantibodies were measured at the University of Bristol, the UK reference laboratory. All radiobinding assays were performed as previously described [2,23]. Samples positive for islet autoantibodies were retested at the second reference laboratory for confirmation. The outcome of islet autoantibody positivity was defined as a positive result at both reference laboratories (confirmed) and the presence of islet autoantibodies (GADA, IA-2A, or IAAs) on 2 or more consecutive visits (persistent). The date of seroconversion to islet autoantibodies (time to first autoantibody) was defined as the date of drawing the first of the 2 consecutive positive samples. The presence of persistent multiple islet autoantibodies was defined as the presence of at least 2 persistent and confirmed islet autoantibodies. The date of persistent multiple islet autoantibodies was defined as the date of drawing the first sample for which the second persistent and confirmed islet autoantibody was detected.

Children with positive islet autoantibodies that were due to maternal IgG transmission were not considered to be positive for that autoantibody unless the child had a negative sample before the first positive sample or the autoantibody persisted beyond 18 months of age [2].

Diabetes was diagnosed according to American Diabetes Association criteria [24].

### Single nucleotide polymorphism typing

In the TEDDY study, single nucleotide polymorphisms (SNPs) of immune-related genes were genotyped using the Illumina ImmunoChip [25]. For SNPs rs11755527 (*BACH2*) and rs689 (*INS*), which were not available on the immunochip, the SNPs rs3757247 (*BACH2*) and rs1004446 (*INS*) were used (S1 Table). No proxy SNPs were available for rs917997 (*IL18RAP*).

### Genetic scores

Genetic scores were determined as described by Winkler et al. [15], without including the intercept value from the logistic regression, and as described by Oram et al. [16]. The Winkler score was originally derived from the Type 1 Diabetes Genetics Consortium case–control dataset, and the Oram score was originally based on the odds ratios available on ImmunoBase (http://www.t1dbase.org/). The genetic score of each individual was derived from weighted values given to the HLA DR3/DR4-DQ8 or DR4-DQ8/DR4-DQ8 genotype plus a weighted value assigned to each susceptible allele of non-HLA SNPs for the Winkler score and HLA class I and non-HLA SNPs for the Oram score (S1 Table). A total of 39/40 non-HLA class II SNPs used in the Winkler score and 26/28 non-HLA class II SNPs used in the Oram score were available to calculate the genetic score in the TEDDY children, while 35/40 and 26/28 SNPs were available for the case–control cohort. For both scores, the HLA DR-DQ genotype weights were added to the weighted risks for each SNP according to the child’s number of risk alleles (0, 1, or 2) for each SNP (S1 Table). Additionally, since the Winkler and Oram scores were derived from partially overlapping genetic loci and each had distinct features, a merged genetic score was derived using the information for all available SNPs contained in the Winkler and Oram scores and was calculated for the TEDDY children (S1 Table). For simplicity, when SNPs overlapped in the Winkler and Oram scores, the mean weight of each SNP in the Winkler and Oram scores was used in the merged score, and for SNPs that were unique in the Winkler or the Oram score, the weight used in the original score was used for the merged score. Exceptions were for 2 SNPs (rs2069763 and rs3825932) that had a negative weight in the Winkler score but a positive weight in the Oram score, where the original Oram score weight was used to calculate the merged score.

### Statistical analyses

An analysis plan was submitted to the TEDDY data coordinating center and approved by the TEDDY steering committee prior to compiling and analyzing the data (S1 Appendix). The merged score was added to this once both the Winkler and Oram scores were found to stratify risk. The Cox analysis and specificity analysis prescribed in the analysis plan were no longer considered to be sufficiently informative to include in the final analysis. The analysis was extended to include type 1 diabetes risk during revision of the manuscript.

For TEDDY children, the cumulative risks of developing islet autoantibodies, multiple islet autoantibodies, and diabetes were estimated using the Kaplan–Meier method and were compared between risk groups using the log-rank test. The risks of islet autoantibodies, multiple islet autoantibodies, and diabetes were calculated for increasing thresholds of the Winkler, Oram, and merged genetic scores. Analyses were also performed after stratification by HLA genotype, geographic location (US, Europe), and sex. The sensitivity of the genetic scores was assessed by calculating the proportion of children who developed islet autoantibodies, multiple islet autoantibodies, and diabetes whose genetic score was above the threshold value. Spearman’s correlation coefficient was used to assess whether the autoantibody risk by age 6 years or diabetes risk by age 10 years—and sensitivity for cases that developed by age 6 years or by age 10 years—changed with increasing score thresholds. The proportion of children in the general population who would be expected to have a genetic score above the threshold was calculated based on the frequency of children with the HLA DR3/DR4-DQ8 or DR4-DQ8/DR4-DQ8 genotype (2.9%) identified in the screening phase of the TEDDY study [22].

For the case–control dataset, we calculated the proportions of non-diabetic controls and cases of type 1 diabetes whose genetic score exceeded the thresholds, with score increments of 0.1. The sensitivity of the genetic scores was assessed by calculating the proportion of cases within the cohort who had a score above the threshold. The empirical risk was calculated as the ratio of the proportion of cases to the proportion of controls above the threshold multiplied by the assumed background risk of 5% for individuals with the DR3/DR4-DQ8 or DR4-DQ8/DR4-DQ8 genotype [11].

The distribution of genetic scores was compared among groups defined by islet autoantibody outcome, geographic location (US, Europe), or sex using the Mann–Whitney *U* test.

All analyses were performed using R 3.3.2 software (R Foundation for Statistical Computing, Vienna, Austria), IBM SPSS version 22.0 (IBM, Armonk, NY), and SAS 9.4 (SAS Institute, Cary, NC).

The datasets generated and analyzed during the current study are available in the NIDDK Central Repository at https://www.niddkrepository.org/studies/teddy. TEDDY immunochip (SNP) data that support the findings of this study have been deposited in NCBI’s Database of Genotypes and Phenotypes (dbGaP) with the primary accession code phs001037.v1.p1.

## Results

### Genetic scores in the case–control population

The Winkler and Oram genetic scores in the WTCCC HLA DR3/DR4-DQ8 or DR4-DQ8/DR4-DQ8 cases were increased as compared to the UK Biobank HLA DR3/DR4-DQ8 or DR4-DQ8/DR4-DQ8 controls (*P <* 0.001; S1 Fig). Using the Winkler score, the calculated actual risk reached 10% above a threshold of 11.72, corresponding to a sensitivity of 58.7% (95% CI 55.2%–62.2%) for the patients who had the HLA DR3/DR4-DQ8 or DR4-DQ8/DR4-DQ8 genotype. Using the Oram score, an actual risk of 10% was reached above a score threshold of 11.67, corresponding to a sensitivity of 36.6% (95% CI 33.2%–40.0%; S1 Fig).

Having verified both scores for type 1 diabetes risk stratification in the case–control dataset, we reasoned that a composite score that included all the features from the Winkler and Oram scores would be justified. We therefore developed a merged genetic score that represented the average weighted values of loci, genotypes, and alleles common to the Winkler and Oram scores, and the original weighted values for loci and alleles that were unique to 1 of the scores (S1 Table). Using the prospectively followed TEDDY cohort, we then asked how well the Winkler, Oram, and merged scores could stratify the risk for pre-symptomatic type 1 diabetes.

### Baseline risk for islet autoantibodies and diabetes in TEDDY children with HLA DR3/DR4-DQ8 or DR4-DQ8/DR4-DQ8 genotype without family history of type 1 diabetes

**Fig 2:**
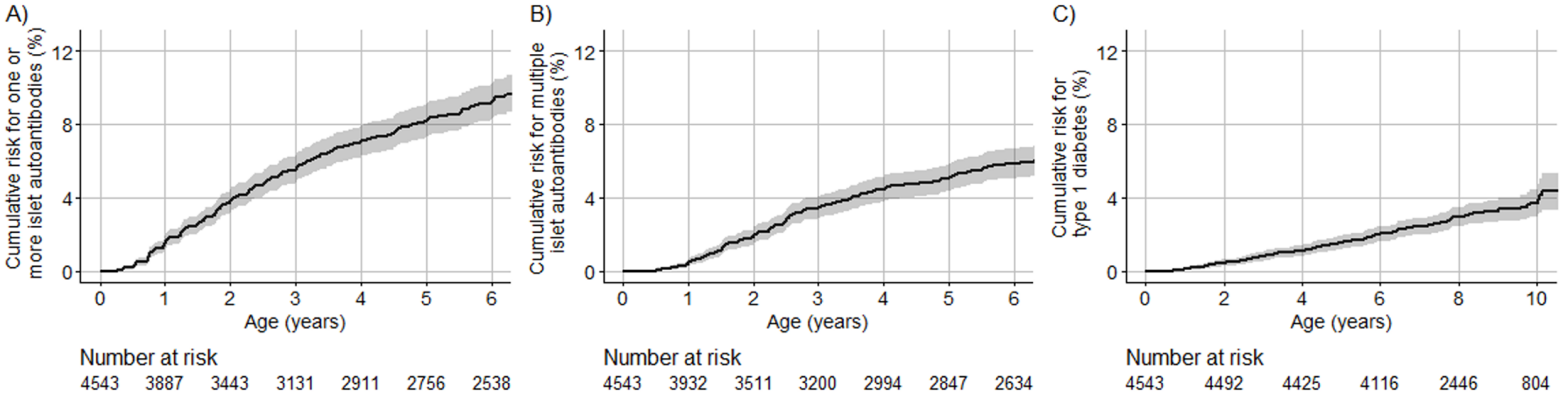
Cumulative risks of 1 or more islet autoantibody, multiple islet autoantibody, and type 1 diabetes in TEDDY children with the HLA DR3/DR4-DQ8 or DR4-DQ8/DR4-DQ8 genotype. The cumulative risk for 1 or more islet autoantibodies (A), multiple islet autoantibodies (B), and type 1 diabetes (C) for TEDDY children (*y-*axis) is shown relative to the age of the children (*x-*axis) and was calculated using the Kaplan–Meier method. The shaded area represents the 95% confidence interval of the cumulative risk. The numbers at risk indicate the number of children included in the analysis at each age.

Seroconversion to islet autoantibodies occurred in 386 children (8.5%) (166 [43.0%] girls), and 4,157 children (91.5%) remained islet autoantibody negative (2,112 [50.8%] girls). Of the 386 children with islet autoantibodies, 241 children (62.4%) developed multiple islet autoantibodies (102 [42.3%] girls; 81 [33.6%] from US). A total of 107 (2.3%) developed diabetes by age 10 years (47 [43.9%] girls). The cumulative risk for developing islet autoantibodies was 9.2% (95% CI 8.2%–10.1%; Fig 2A), of developing multiple islet autoantibodies (pre-symptomatic type 1 diabetes) by age 6 years was 5.8% (95% CI 5.0%–6.6%; Fig 2B), and of developing diabetes by age 10 years was 3.7% (95% CI 3.0%–4.4%; Fig 2C).

### Genetic scores in TEDDY children

**Fig 3:**
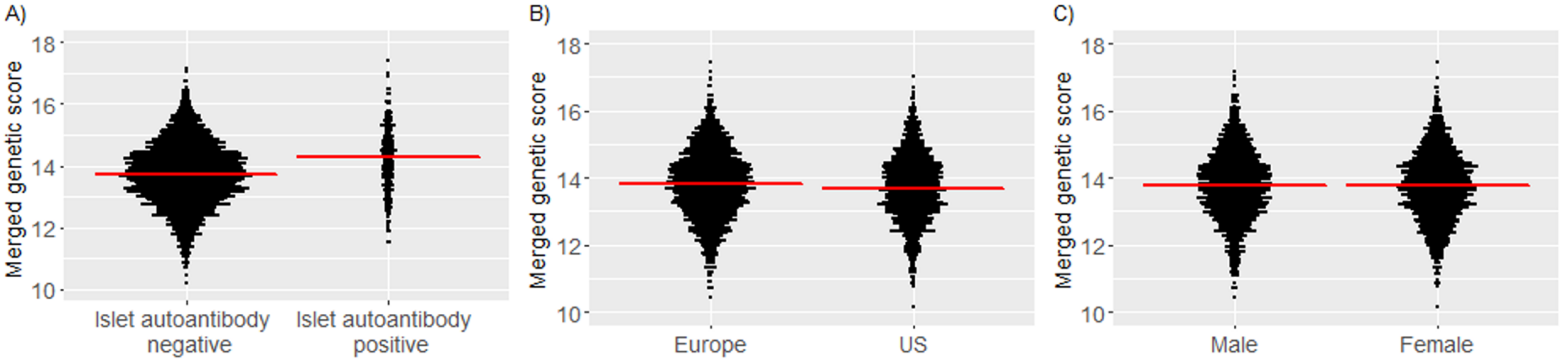
Merged genetic score in TEDDY children according to their islet autoantibody outcome, geographic location, and sex. Islet autoantibody outcome (A); geographic location (B); sex (C). Red horizontal lines indicate the median genetic score value in each group.

We examined whether Winkler, Oram, and merged genetic scores were increased in children who developed islet autoantibodies. The genetic scores were calculated in 3,498 (1,471 US) children who had material for additional genetic analysis. The median follow-up in these children was 7.39 years. For each of the Winkler, Oram, and merged scores, the score was greater in children who developed islet autoantibodies by 6 years of age as compared to children who remained islet autoantibody negative (*P <* 0.001; Fig 3A and S2 Fig). The median merged score was 14.3 (IQR, 13.6–14.9) in children who developed islet autoantibodies versus 13.7 (IQR, 13.1–14.4) in children who remained islet autoantibody negative. The genetic scores were also slightly greater in European children (median merged score, 13.8; IQR, 13.1–14.5) than in US children (13.7; IQR, 13.1–14.4; *P =* 0.003; Fig 3B and S2 Fig). The frequencies of minor alleles differed between the US and European children for 7 of 43 SNPs (Bonferroni-corrected *P* of 0.05/43 = 0.0012; S2 Table). Scores were not different between boys and girls (*P =* 0.69; Fig 3C and S2 Fig).

### Risk for islet autoantibodies and diabetes according to the genetic scores

**Fig 4:**
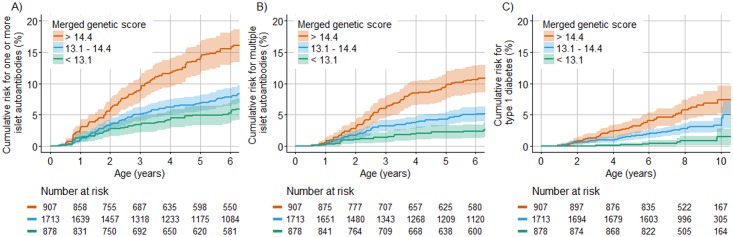
Cumulative risks of 1 or more islet autoantibody, multiple islet autoantibody, and type 1 diabetes development in TEDDY children with the HLA DR3/DR4-DQ8 or DR4-DQ8/DR4-DQ8 genotype stratified by their merged score. The cumulative risk of developing 1 or more islet autoantibodies (A), multiple islet autoantibodies (B), and type 1 diabetes (C) (*y-*axis) is shown relative to age in years (*x-*axis) and was calculated using the Kaplan–Meier method. Curves are shown for children with genetic scores in the upper (orange line), lower (green line), and 2 middle (blue line) quartiles. The shaded areas represent the 95% confidence interval of the cumulative risk. The numbers at risk indicate the number of children included in the analysis at each age.

We next asked if and how much the genetic scores could stratify risk in TEDDY children without a family history of type 1 diabetes. To address this, the cumulative risk for developing islet autoantibodies and for diabetes was compared between HLA DR3/DR4-DQ8 and DR4-DQ8/DR4-DQ8 children who were in the upper quartile, middle 2 quartiles, and lower quartile of the merged genetic score (Fig 4). The cumulative risk for developing islet autoantibodies by 6 years of age was 16.0% (95% CI 13.3%–18.6%) among children with a merged genetic score of >14.4, representing the upper quartile, compared with 6.9% (95% CI 5.9%–8.0%) in children with a score of ≤14.4 (*P <* 0.001). The cumulative risk for developing multiple islet autoantibodies by 6 years of age was 11.0% (95% CI 8.7%–13.3%) in children with a score of >14.4, compared with 4.1% (95% CI 3.3%–4.9%) in children with a score of ≤14.4 (*P <* 0.001). The cumulative risk for developing diabetes by age 10 years was 7.6% (95% CI 5.3%–9.9%) in children with a score of >14.4, compared with 2.7% (95% CI 1.9%–3.6%) in children with a score of ≤14.4 (*P <* 0.001). The risks were also stratified by the Winkler and Oram scores (*P <* 0.001; S3 Fig). However, the merged genetic score performed better than both the Winkler and Oram scores in identifying the HLA DR3/DR4-DQ8 and HLA DR4-DQ8/DR4-DQ8 children who developed multiple islet autoantibodies (S4 Fig).

The merged genetic score stratified the risk for islet and multiple islet autoantibodies and for diabetes both in children who had the HLA DR3/DR4-DQ8 genotype and in children who had the HLA DR4-DQ8/DR4-DQ8 genotype (S5 Fig). The risks of islet autoantibodies, multiple islet autoantibodies, and diabetes in children with a merged genetic score of >14.4 were not significantly different between US and European children (*P =* 0.16, *P =* 0.97, and P = 0.96, respectively; S6 Fig), but autoantibody risks were higher in boys than in girls (*P =* 0.001 for 1 or more islet autoantibodies and *P =* 0.01 for multiple islet autoantibodies; S6 Fig).

### Sensitivity versus risk for islet autoantibodies and diabetes according to the merged genetic score

**Fig 5:**
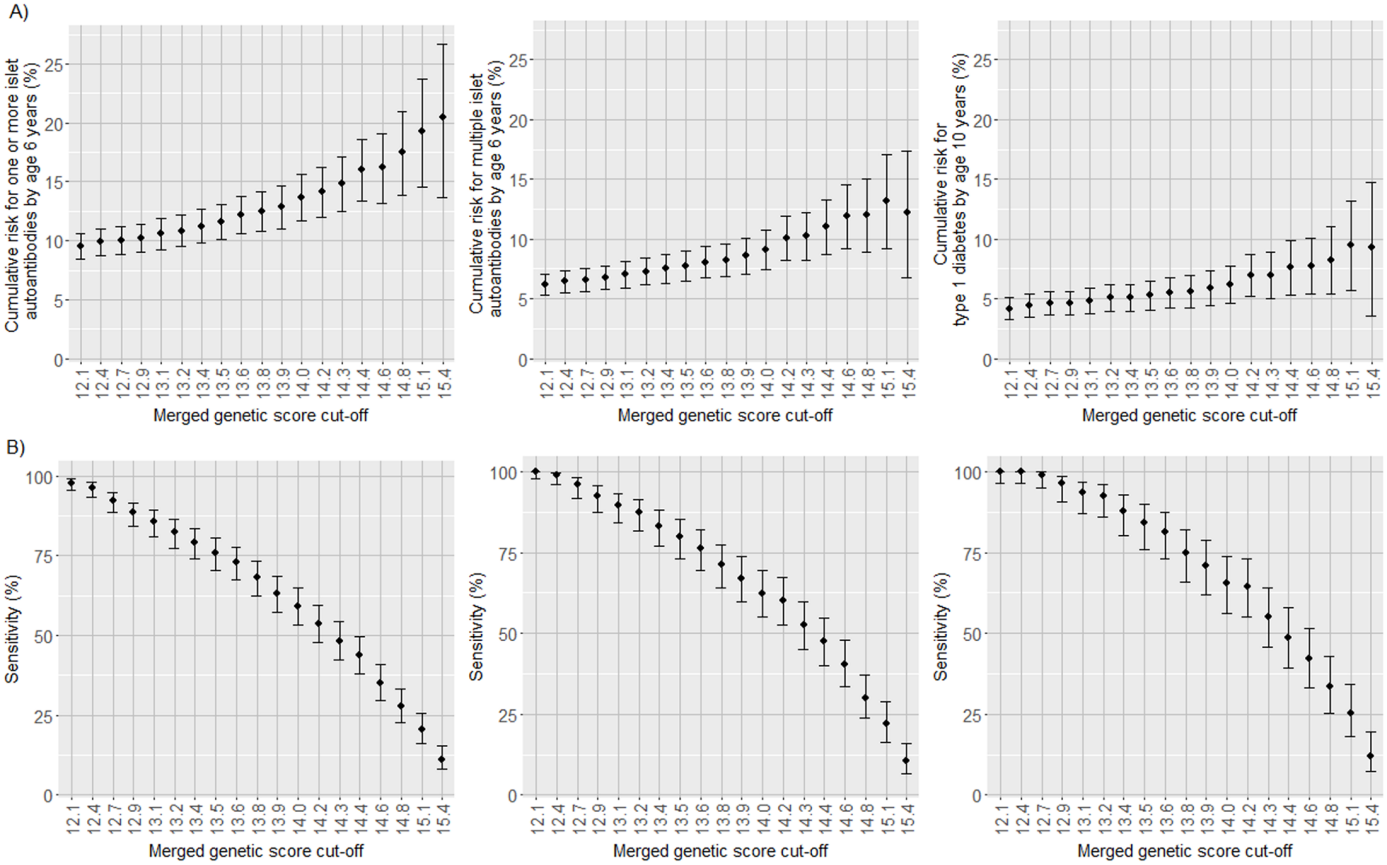
Cumulative risks and the proportion of cases identified for 1 or more islet autoantibodies, multiple islet autoantibodies, and type 1 diabetes in TEDDY children with the HLA DR3/DR4-DQ8 or DR4-DQ8/DR4-DQ8 genotype according to increasing thresholds of the merged genetic score. Cumulative risk for developing islet autoantibodies by age 6 years and diabetes by age 10 years (A) and the proportion of cases positive for islet autoantibodies by age 6 years and diabetes by age 10 years (sensitivity; B) in TEDDY children with the HLA DR3/DR4-DQ8 or DR4-DQ8/DR4-DQ8 genotype stratified by their merged genetic score. The risk and sensitivity are shown for each increment in the genetic score by the 5th percentile of scores in the TEDDY children, ranging from >12.1 (the 5th percentile of children) to >15.4 (the 95th percentile of children). The risk and sensitivity are shown for the development of 1 or more islet autoantibodies (left panels), multiple islet autoantibodies (middle panels), and type 1 diabetes (right panels). Error bars indicate 95% confidence intervals.

Islet autoantibody and diabetes risk appeared incremental with increasing merged genetic score. Since the efficiency of a test is measured by both sensitivity and positive predictive value (risk), we examined the relationship between sensitivity and risk at increasing thresholds for the genetic score (Fig 5; S3–S5 Tables). As predicted, the cumulative risk for developing islet autoantibodies or multiple islet autoantibodies by age 6 years and diabetes by age 10 years increased (*P <* 0.001) and the sensitivity decreased (*P <* 0.001) with each increment in the genetic score threshold by the 5th percentile of the cohort. The risk for multiple islet autoantibodies reached a maximum of 13.2% (95% CI 9.2%–17.1%) above a threshold of >15.1, which identified 38 of the 173 children who developed multiple islet autoantibodies by age 6 years, corresponding to a sensitivity of 22.0% (95% CI 16.4%–28.7%). Above a threshold of 14.4, representing the upper 25% of merged genetic score values and where risk for multiple islet autoantibodies was 11.0% (95% CI 8.7%–13.3%), 82 of the 173 children who developed multiple islet autoantibodies by age 6 years were identified (sensitivity, 47.4%; 95% CI 40.1%–54.8%). In a population where the prevalence of HLA DR3/DR4-DQ8 and HLA DR4-DQ8/DR4-DQ8 genotypes is similar to that of the TEDDY cohort (2.9%), using the threshold of 14.4 would identify <1% of all newborns without a prior family history of type 1 diabetes.

## Discussion

Genetic scores derived from logistic regression of numerous loci associated with type 1 diabetes susceptibility were able to stratify the risk for pre-symptomatic and clinical type 1 diabetes in a prospective cohort of children with high-risk HLA genotypes but no family history of type 1 diabetes. The risks of developing islet autoantibodies, multiple islet autoantibodies, and diabetes increased with each increment in the genetic score. A genetic score that would identify <1% of all newborn infants was associated with a risk for developing multiple islet autoantibodies of >10% by 6 years of age. This compares to a background population risk of around 0.4% [26]. These findings provide a paradigm for identifying infants whose risk for developing type 1 diabetes is more than 25 times that of the general population, twice that of infants identified by the highest-risk HLA genotypes alone, and higher than that of children with a first-degree relative with type 1 diabetes.

The study was performed using a large number of children who were prospectively followed for the development of islet autoantibodies from infancy. The findings were consistent between US and European children and for 2 independently derived genetic scores that were used to develop the merged genetic score. We note, however, that the risk scores were generated using the outcome of clinical, or stage 3, type 1 diabetes. It has been previously noted in multiple studies, including TEDDY, that many, but not all of the genes associated with type 1 diabetes confer risk for islet autoantibodies [25,27]. For this reason, the performance of a genetic score for identifying pre-symptomatic type 1 diabetes might improve if genes and weights for islet autoantibody susceptibility are incorporated into the score. Genetic score performance may also be improved if more accurate estimates of risk weight for homozygous versus heterozygous alleles were available. Of note, the score does not include all type 1 diabetes susceptibility genes and does not contain weights for several HLA class I alleles that confer susceptibility for type 1 diabetes [28–30]. Finally, the current genetic scores were derived from cohorts of mostly individuals of European descent, and it is likely that the genetic scores may not be suitable for all races or ethnic groups.

The study was performed to extend the opportunities for early identification of individuals at increased risk for disease. Previous primary prevention trials in type 1 diabetes involved HLA typing of infants with a family history of type 1 diabetes [3,4,31]. The enrollment of participants into these trials took several years, and the proportion of all cases of childhood type 1 diabetes that were represented by the inclusion criteria was less than 5%, limiting the generalizability of trial outcomes. Screening that is limited to HLA typing of the general population can identify individuals with 3% to 5% risk, which may be insufficient for enrollment into primary prevention studies in which infants are exposed to treatment. Indeed, the TRIGR study used a combination of HLA typing and family history in order to identify infants whose risk for type 1 diabetes was 10% [3]. Therefore, we set a risk target of 10%, which was achieved in our study when we used the development of multiple islet autoantibodies as a marker for pre-symptomatic type 1 diabetes. The risk threshold was reached when the Winkler, Oram, or merged genetic score was used in children with the 2 highest-risk HLA genotypes, DR3/DR4-DQ8 and DR4-DQ8/DR4-DQ8, which can be detected by typing 3 SNPs. In a European population, these 2 genotypes were present in around 40% of all cases of childhood type 1 diabetes [11]. The merged genetic score threshold of >14.4 identified almost 50% of children with these genotypes who developed multiple islet autoantibodies or diabetes. Therefore, we surmise that our risk score threshold would identify up to 20% of children without family history of type 1 diabetes who will develop the disease. Moreover, the screening strategy is relatively inexpensive, and is now being used in the Primary Oral Insulin Trial (https://clinicaltrials.gov/ct2/show/NCT03364868), where DNA extraction from blood spots and typing are performed for less than US$8 per sample. Extending the strategy to individuals with other HLA genotypes is possible, but the other genotypes are less frequent in type 1 diabetes and are associated with a lower risk than that conferred by the DR3/DR4-DQ8 and DR4-DQ8/DR4-DQ8 genotypes. Therefore, the inclusion of other genotypes is unlikely to further improve risk stratification.

In conclusion, a genetic score based on 3 SNPs for HLA class II genotyping and 41 SNPs in other genes identified <1% of newborn children who, in the absence of a family history of type 1 diabetes, had a >10% risk for developing multiple islet autoantibodies by 6 years of age. This greatly extends the possibilities of enrolling participants into clinical trials aimed at evaluating type 1 diabetes prevention strategies that could be applied in infancy and before the development of autoimmunity [32].

## Supporting information

S1 Appendix TEDDY manuscript proposal submission form. (DOC)

S1 Fig Genetic scores and estimated risk for type 1 diabetes in the cases and controls with the HLA DR3/DR4-DQ8 or DR4-DQ8/DR4-DQ8 genotype. Genetic scores calculated using the Winkler model (left panels) and the Oram model (right panels) in the UK Biobank and Wellcome Trust Case Control Consortium (WTCCC) controls, and in WTCCC cases with the HLA DR3/DR4-DQ8 or DR4-DQ8/DR4-DQ8 genotype (A). The empirically calculated risk of type 1 diabetes (*y-*axis) and the proportion of all cases of type 1 diabetes in each cohort (*x-*axis) are shown for both genetic scores (B).
(TIF)

S2 Fig Winkler and Oram genetic scores in TEDDY children according to their islet autoantibody outcome, geographic location, and sex. Islet autoantibody outcome (A); geographic location (B); sex (C). Red horizontal lines indicate the median genetic score value in each group.
(TIF)

S3 Fig Cumulative risks of 1 or more islet autoantibody, multiple islet autoantibody, and type 1 diabetes development in TEDDY children with the HLA DR3/DR4-DQ8 or DR4-DQ8/DR4-DQ8 genotype stratified by their Winkler and Oram score. Cumulative risks of developing 1 or more islet autoantibodies (A, B), multiple islet autoantibodies (C, D), and type 1 diabetes (E, F) in TEDDY children with the HLA DR3/DR4-DQ8 or DR4-DQ8/DR4-DQ8 genotype stratified by their Winkler (A, C, E) and Oram (B, D, F) genetic scores. The risk (*y-*axis) is shown relative to the age in years (*x-*axis) and was calculated using the Kaplan–Meier method. Curves are shown for children with genetic scores in the upper (orange line), lower (green line), and 2 middle (blue line) quartiles. The shaded areas represent the 95% confidence interval of the cumulative risk. The numbers at risk indicate the number of children included in the analysis at each age.
(TIF)

S4 Fig Time-dependent discrimination accuracy of the genetic scores to identify TEDDY children who developed multiple islet autoantibodies. Three scores are compared (RO = Oram score, WI = Winkler score, ME = merged score). (A) We calculated the integral of a time-dependent receiver operating characteristic curve [33], indicated on the *y-*axis for each genetic risk score from 1 year to 10 years with increments of 100 days. (B) To obtain a distribution for each of these predicted scores, we performed 2,000 paired bootstrap analyses for each genetic risk score, with the results shown as box pots (diamonds indicate the integrated area under the curve [AUC] for the full TEDDY data). These bootstrap analyses were further used to assess statistical differences of the time-dependent receiver operating characteristic curve estimates per genetic risk score. To this end, we calculated Bayes factors of the paired estimates [34] of 2 risk scores. Specifically, the Bayes factor of risk score 1 (RS1) versus risk score 2 (RS2) is calculated as the posterior probability of the alternative hypothesis (RS1 is better than RS2), defined as the fraction of bootstrap analyses in which RS1 is better than RS2, divided by the posterior probability of the null hypothesis (RS1 is no better than RS2), defined as the fraction of bootstrap analyses in which RS1 is no better than RS2. We denoted the merged genetic score as superior to the Winkler score (Bayes factor = 6.2) and Oram score (Bayes factor = 94), and no difference between the Winkler score and Oram score, with a Bayes factor of 1.2 [35].
(TIF)

S5 Fig Cumulative risks of 1 or more islet autoantibody, multiple islet autoantibody, and type 1 diabetes development in TEDDY children with the HLA DR3/DR4-DQ8 or DR4-DQ8/DR4-DQ8 genotype stratified by their merged genetic score and their HLA genotype. Cumulative risks of developing 1 or more islet autoantibodies (A, B), multiple islet autoantibodies (C, D), and type 1 diabetes (E, F) in TEDDY children with the HLA DR3/DR4-DQ8 (A, C, E) or DR4-DQ8/DR4-DQ8 (B, D, F) genotype. The risk (*y-*axis) is shown relative to the age in years (*x-*axis) and was calculated using the Kaplan–Meier method. Curves are shown for children with merged genetic scores in the upper (orange line), lower (green line), and 2 middle (blue line) quartiles. The shaded areas represent the 95% confidence interval of the cumulative risk. The numbers at risk indicate the number of children included in the analysis at each age.
(TIF)

S6 Fig Cumulative risks of 1 or more islet autoantibody, multiple islet autoantibody, and type 1 diabetes development in TEDDY children with the HLA DR3/DR4-DQ8 or DR4-DQ8/DR4-DQ8 genotype stratified by geographic location and sex. Cumulative risks of the development of 1 or more islet autoantibodies (A, B), multiple islet autoantibodies (C, D), and type 1 diabetes (E, F) in TEDDY children with the HLA DR3/DR4-DQ8 or DR4-DQ8/DR4-DQ8 genotype and merged genetic score > 14.4. The risk (*y-*axis) is shown relative to the age in years (*x-*axis) and was calculated using the Kaplan–Meier method. Curves are shown for children divided by geographic location (A, C, E; Europe, yellow lines; US, green lines) and sex (B, D, F; boys, blue lines; girls, red lines). The shaded areas represent the 95% confidence interval of the cumulative risk. The numbers at risk indicate the number of children included in the analysis at each age.
(TIF)

S1 GRIPS Statement (DOC)

S1 Table Weights for single nucleotide polymorphisms used to calculate the genetic scores. (DOC)

S2 Table Frequencies of risk alleles in TEDDY children with the HLA DR3/DR4-DQ8 or DR4-DQ8/DR4-DQ8 genotype. (DOC)

S3 Table Risk of developing 1 or more islet autoantibodies by age 6 years and the proportion of cases positive for any islet autoantibodies (sensitivity) in TEDDY children with the HLA DR3/DR4-DQ8 or DR4-DQ8/DR4-DQ8 genotype stratified by their merged genetic score, with corresponding 95% confidence intervals. (DOC)

S4 Table Risk of developing multiple islet autoantibodies by age 6 years and the proportion of cases positive for multiple islet autoantibodies (sensitivity) in TEDDY children with the HLA DR3/DR4-DQ8 or DR4-DQ8/DR4-DQ8 genotype stratified by their merged genetic score, with corresponding 95% confidence intervals. (DOC)

S5 Table Risk of developing type 1 diabetes by age 10 years and the proportion of cases positive for type 1 diabetes (sensitivity) in TEDDY children with the HLA DR3/DR4-DQ8 or DR4-DQ8/DR4-DQ8 genotypes stratified by their merged genetic score, with corresponding 95% confidence intervals. (DOC)

## Abbreviations

GADA: glutamic acid decarboxylase antibody
HLA: human leukocyte antigen
IA-2A: insulinoma antigen-2 antibody
IAA: insulin autoantibody
TEDDY: The Environmental Determinants of Diabetes in the Young
WTCCC: Wellcome Trust Case Control Consortium
