# Supplementary material for: Genetic scores to stratify risk of developing multiple islet autoantibodies and type 1 diabetes: A prospective study in children
Source: PLoS Med. 2018 Apr 3;15(4):e1002548. doi: 10.1371/journal.pmed.1002548 (PMC5882115; doi:10.1371/journal.pmed.1002548)
Supplement: S1 Appendix — (DOC) [file pmed.1002548.s001.doc]

## TEDDY Manuscript Proposal Submission Form

Date Submitted: 2-10-2016

- 1. **Brief Background and Aim:**

More than 40 regions of the human genome confer susceptibility for type 1 diabetes. We recently have identified a weighed set of HLA and non-HLA SNP combinations which improved prediction of type 1 diabetes over that provided by HLA alone (1). Similarly, the group of Michael Weedon, Richard Oram, and Andrew T Hattersley identified 30 SNPs that discriminate effectively between type 1 and type 2 diabetes in the Welcome Trust Case Control Consortium (2).

- 1. **Research Hypotheses:**

We hypothesize that our weighted risk model can improve prediction of beta cell autoimmunity (defined by multiple islet autoantibodies) in TEDDY children and therefore could be helpful in identifying children with up to 10% risk for persistent confirmed beta cell autoimmunity from the general population. This would help the design of primary prevention RCTs in the future.

- 1. **Data** [Identify population to be used, variables to be used, and inclusions and exclusions]

1. Variables to be used:

HLA genotype (including HLA DRB1*04 subtype)

SNPs from the Munich and Exeter publications (see table 1)

Islet autoantibody status (no, single, multiple)

Date/age of seroconversion (first islet autoantibody)

Follow-up time from birth to last sample

Sex, male, female

Country: USA, Sweden, Finland, Germany

Table A: SNPs to be used for analysis

|  |  |
| --- | --- |
| rs2476601 | ptpn22 |
| rs689 | ins |
| rs12722495 | il2ra |
| rs2292239 | erbb3 |
| rs2290400 | ormdl3 |
| rs425105 | prkd2 |
| rs3024505 | il10 |
| rs11755527 | bach2 |
| rs4788084 | il27 |
| rs3184504 | sh2b3 |
| rs7020673 | glis3 |
| rs10509540 | rnls |
| rs3788013 | ubash3a |
| rs3825932 | ctsh |
| rs5753037 |  |
| rs10517086 |  |
| rs6897932 | il7r |
| rs229541 | il2b |
| rs1990760 | ifih1 |
| rs2816316 | rgs1 |
| rs7804356 | scap2 |
| rs6920220 | tnfaip3 |
| rs947474 | prkcq |
| rs7221109 |  |
| rs917997 | il18 |
| rs2664170 | gab3 |
| rs1465788 | zfp3l1 |
| rs3087243 | ctla4 |
| rs7202877 |  |
| rs9388489 | c6orf173 |
| rs763361 | cd226 |
| rs5979785 | tlr8 |
| rs4948088 | cobl |
| rs12708716 | CLEC16A |
| rs4763879 | cd69 |
| rs1738074 | tagap |
| rs2281808 | sirpg |
| rs4900384 |  |
| rs45450798 | ptpn2 |
| rs2069763 | il2 |
| rs1264813 | HLA_A_24 |
| rs2395029 | HLA_B_5701 |
| rs10509540 | C10orf59 |
| rs11594656 | IL2RA |
| rs17574546 |  |

1. Inclusions:

Teddy children who are GP and have the DR3/4 or the DR4/4 genotypes (the DR3/3 genotype is not considered because we do not expect to identify children with 10% or higher risk in this group using the algorithms that will be applied)

1. Exclusions: none
   1. **Analysis plan and methods** *[Provide details of proposed statistical analyses. Please consult with statistician assigned to this proposal when developing this section.]***:**

The aim is to determine whether genetic algorithms are able to identify GP children who have a risk approaching 10% for the development of multiple beta cell autoantibodies in childhood. Outcome risk will be estimated by Kaplan Meier analysis.

Refined risk scores from the Winkler and the Oram studies will be applied to the children. We will then categorize the children into 4 quartiles based on the risk scores. We propose that the reference category be the combined second and third quartiles (25^th^ to 75^th^ centiles) and that the lowest risk score category and the highest risk score categories are compared to the reference using COX survival analysis. Confounders should include country, sex.

Reporting would be:

1. the multiple islet autoantibody risk by age 4,5,6 years for each of the 4 quartiles for each of the two algoirthms
2. the HRs for lowest and highest quartile of scores (again each of the two algorithms).
3. Sensitivity and specificity for each of the 4 quartiles for each of the two algorithms.

As a sub-analysis, we would restrict the above analysis to the DR3/4 children.

A second analysis is an ROC curve where outcome is defined as multiple beta cell autoantibodies by age 5 years (yes, no) and the genetic risk score is used as the variable (performed for each of the two algorithms).

We will also apply the risk scores to the UK Biobank data (120,000 individuals) in order to estimate the frequency of the population with the risk score above the 75^th^ centile of the TEDDY GP HLA DR3/4, DR4/4 children in order to model recruitment to a trial.

- 1. **References:**

1. Winkler C, Krumsiek J, Lempainen J, Achenbach P, Grallert H, Giannopoulou E et al. A strategy for combining minor genetic susceptibility genes to improve prediction of disease in type 1 diabetes. Genes Immun. 2012;13(7):549-555
2. Oram RA, Patel K, Hill A, Shields B, McDonald TJ, Jones A et al. A type 1 diabetes genetic risk score can aid discrimination between type 1 and type 2 diabetes in young adults. Diabetes Care. 2016;39(3):337–344
