# Supplementary figures and images for: Genetic scores to stratify risk of developing multiple islet autoantibodies and type 1 diabetes: A prospective study in children
Source: PLoS Med. 2018 Apr 3;15(4):e1002548. doi: 10.1371/journal.pmed.1002548 (PMC5882115; doi:10.1371/journal.pmed.1002548)

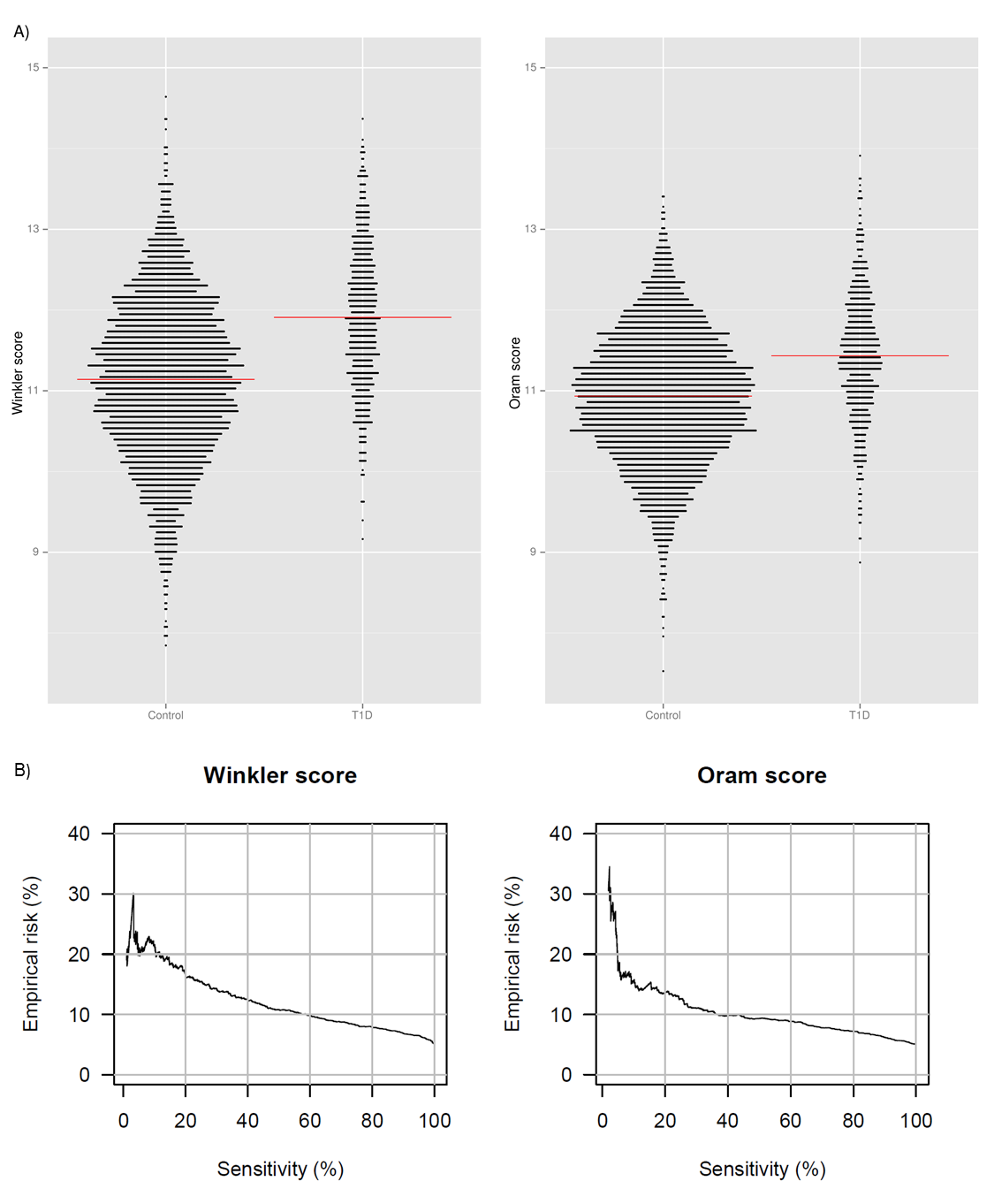

Supplement: S1 Fig — Genetic scores calculated using the Winkler model (left panels) and the Oram model (right panels) in the UK Biobank and Wellcome Trust Case Control Consortium (WTCCC) controls, and in WTCCC cases with the HLA DR3/DR4-DQ8 or DR4-DQ8/DR4-DQ8 genotype (A). The empirically calculated risk of type 1 diabetes (y-axis) and the proportion of all cases of type 1 diabetes in each cohort (x-axis) are shown for both genetic scores (B). (TIF) [file pmed.1002548.s002.tif]

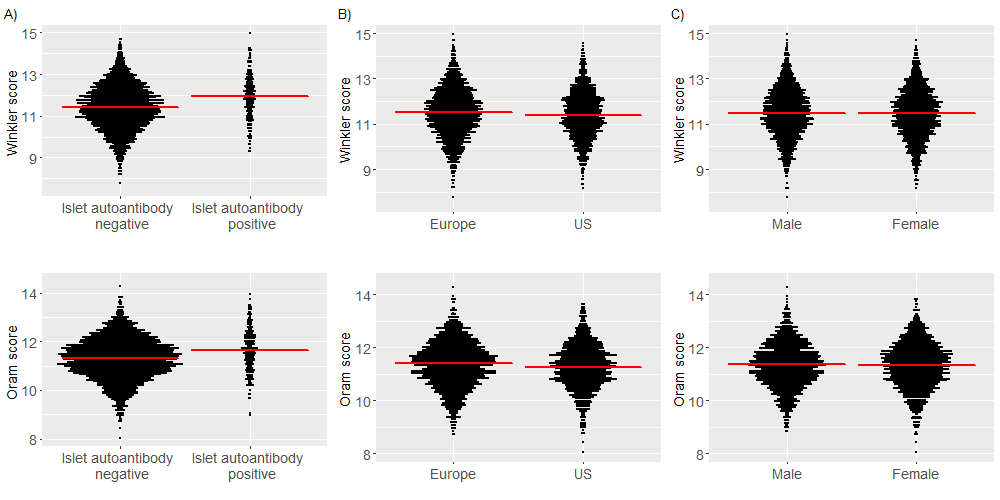

Supplement: S2 Fig — Islet autoantibody outcome (A); geographic location (B); sex (C). Red horizontal lines indicate the median genetic score value in each group. (TIF) [file pmed.1002548.s003.tif]

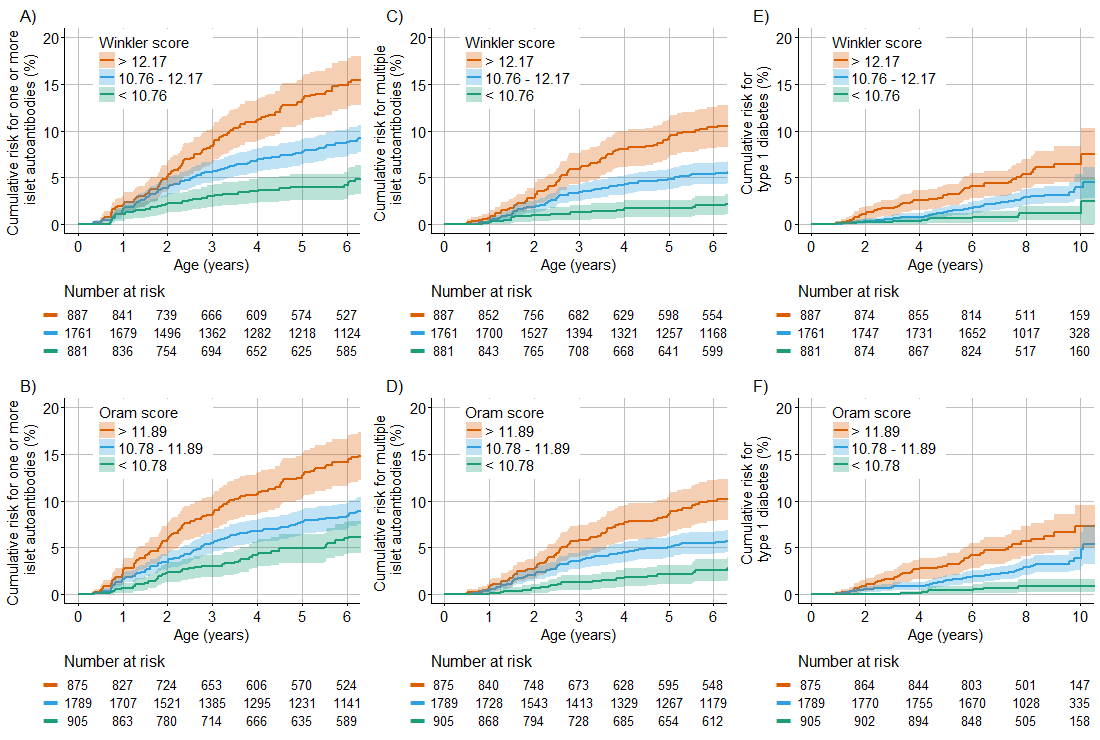

Supplement: S3 Fig — Cumulative risks of developing 1 or more islet autoantibodies (A, B), multiple islet autoantibodies (C, D), and type 1 diabetes (E, F) in TEDDY children with the HLA DR3/DR4-DQ8 or DR4-DQ8/DR4-DQ8 genotype stratified by their Winkler (A, C, E) and Oram (B, D, F) genetic scores. The risk (y-axis) is shown relative to the age in years (x-axis) and was calculated using the Kaplan–Meier method. Curves are shown for children with genetic scores in the upper (orange line), lower (green line), and 2 middle (blue line) quartiles. The shaded areas represent the 95% confidence interval of the cumulative risk. The numbers at risk indicate the number of children included in the analysis at each age. (TIF) [file pmed.1002548.s004.tif]

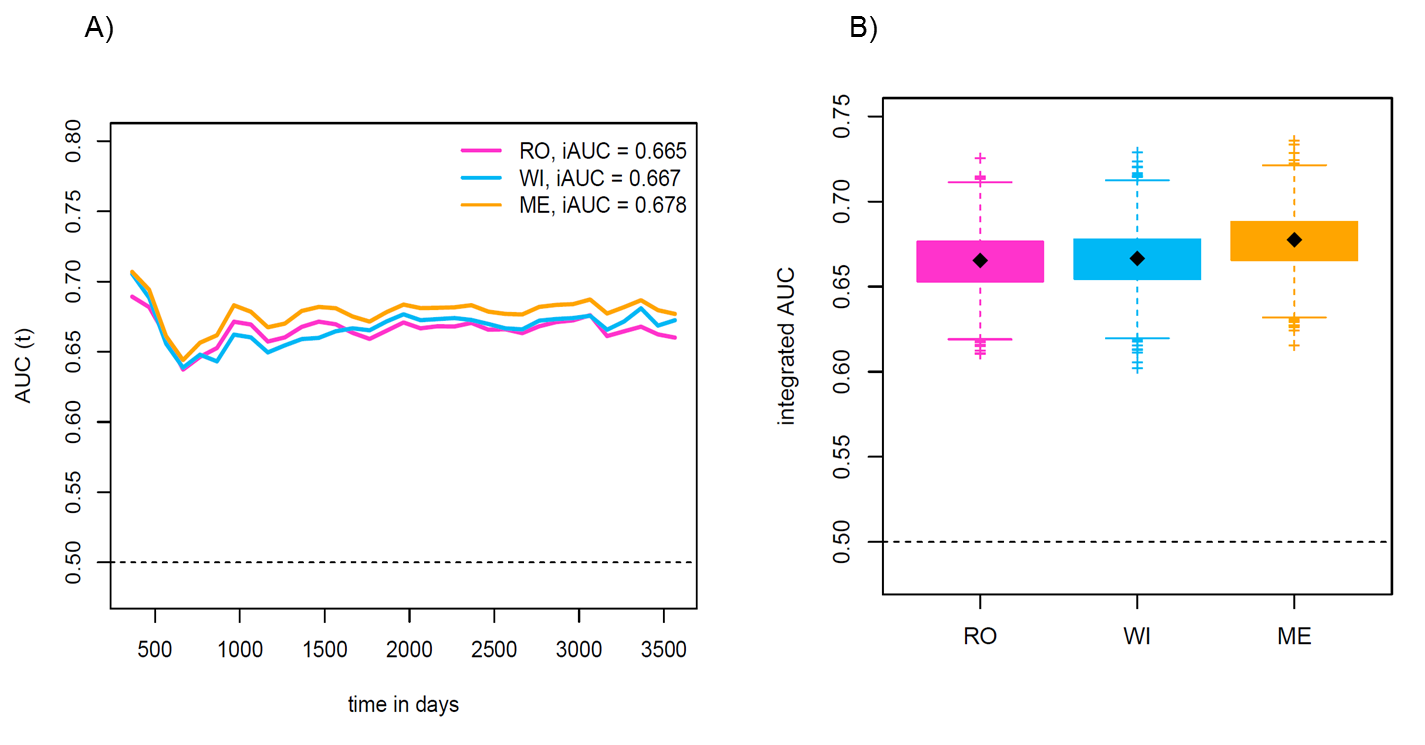

Supplement: S4 Fig — Three scores are compared (RO = Oram score, WI = Winkler score, ME = merged score). (A) We calculated the integral of a time-dependent receiver operating characteristic curve [33], indicated on the y-axis for each genetic risk score from 1 year to 10 years with increments of 100 days. (B) To obtain a distribution for each of these predicted scores, we performed 2,000 paired bootstrap analyses for each genetic risk score, with the results shown as box pots (diamonds indicate the integrated area under the curve [AUC] for the full TEDDY data). These bootstrap analyses were further used to assess statistical differences of the time-dependent receiver operating characteristic curve estimates per genetic risk score. To this end, we calculated Bayes factors of the paired estimates [34] of 2 risk scores. Specifically, the Bayes factor of risk score 1 (RS1) versus risk score 2 (RS2) is calculated as the posterior probability of the alternative hypothesis (RS1 is better than RS2), defined as the fraction of bootstrap analyses in which RS1 is better than RS2, divided by the posterior probability of the null hypothesis (RS1 is no better than RS2), defined as the fraction of bootstrap analyses in which RS1 is no better than RS2. We denoted the merged genetic score as superior to the Winkler score (Bayes factor = 6.2) and Oram score (Bayes factor = 94), and no difference between the Winkler score and Oram score, with a Bayes factor of 1.2 [35]. (TIF) [file pmed.1002548.s005.tif]

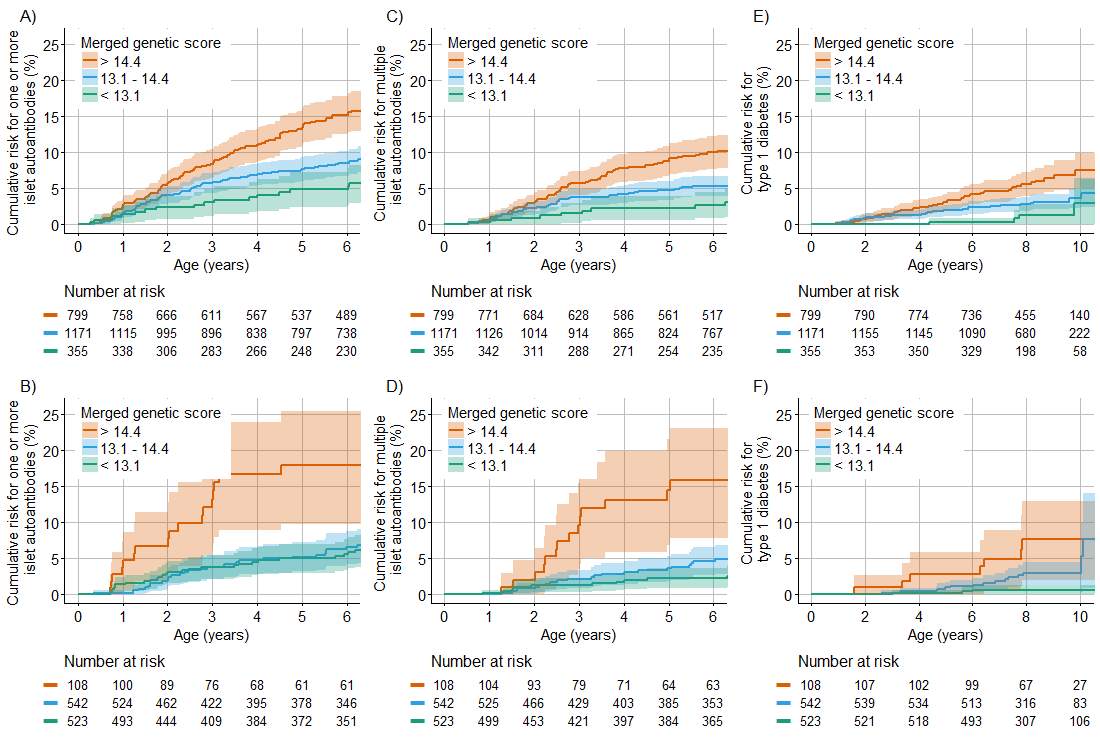

Supplement: S5 Fig — Cumulative risks of developing 1 or more islet autoantibodies (A, B), multiple islet autoantibodies (C, D), and type 1 diabetes (E, F) in TEDDY children with the HLA DR3/DR4-DQ8 (A, C, E) or DR4-DQ8/DR4-DQ8 (B, D, F) genotype. The risk (y-axis) is shown relative to the age in years (x-axis) and was calculated using the Kaplan–Meier method. Curves are shown for children with merged genetic scores in the upper (orange line), lower (green line), and 2 middle (blue line) quartiles. The shaded areas represent the 95% confidence interval of the cumulative risk. The numbers at risk indicate the number of children included in the analysis at each age. (TIF) [file pmed.1002548.s006.tif]

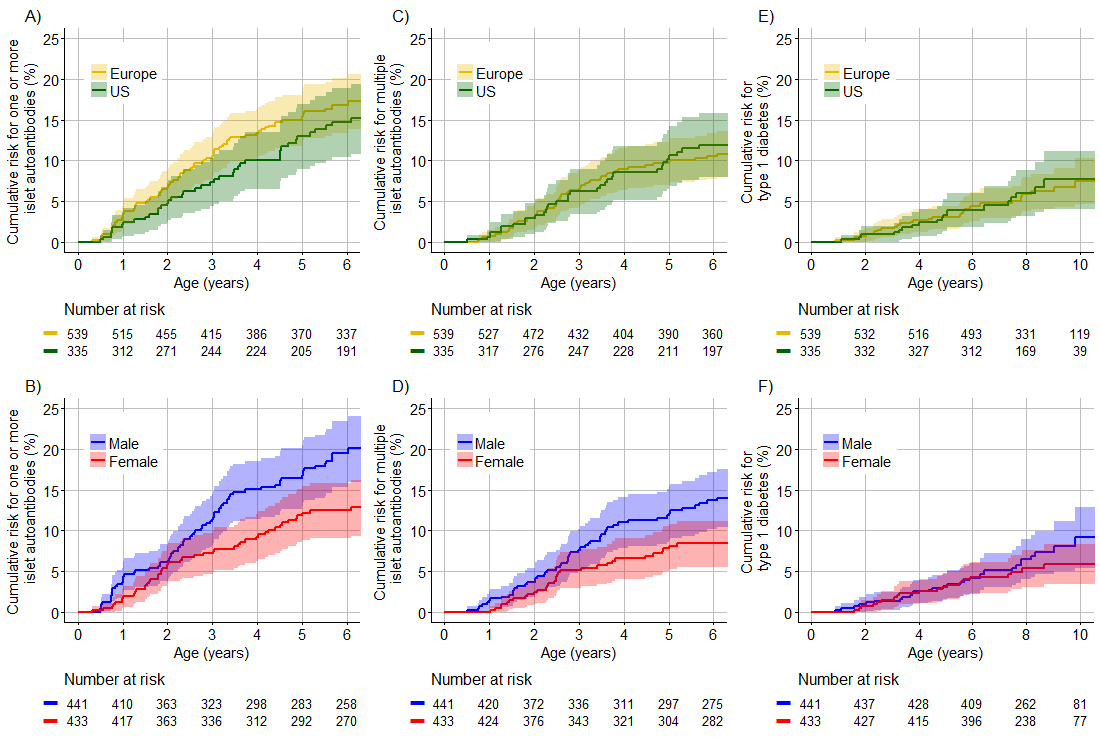

Supplement: S6 Fig — Cumulative risks of the development of 1 or more islet autoantibodies (A, B), multiple islet autoantibodies (C, D), and type 1 diabetes (E, F) in TEDDY children with the HLA DR3/DR4-DQ8 or DR4-DQ8/DR4-DQ8 genotype and merged genetic score > 14.4. The risk (y-axis) is shown relative to the age in years (x-axis) and was calculated using the Kaplan–Meier method. Curves are shown for children divided by geographic location (A, C, E; Europe, yellow lines; US, green lines) and sex (B, D, F; boys, blue lines; girls, red lines). The shaded areas represent the 95% confidence interval of the cumulative risk. The numbers at risk indicate the number of children included in the analysis at each age. (TIF) [file pmed.1002548.s007.tif]
