# Supplementary material for: Genetic scores to stratify risk of developing multiple islet autoantibodies and type 1 diabetes: A prospective study in children
Source: PLoS Med. 2018 Apr 3;15(4):e1002548. doi: 10.1371/journal.pmed.1002548 (PMC5882115; doi:10.1371/journal.pmed.1002548)
Supplement: S1 GRIPS Statement — (DOC) [file pmed.1002548.s008.doc]

| **TITLE & ABSTRACT** | | |
| --- | --- | --- |
|  | 1 | (a) Identify the article as a study of risk prediction using genetic factors. (b) Use recommended keywords in the abstract: genetic or genomic, risk, prediction.  Title contains genetic and risk. Abstract (page 3)contains genetic, risk, and prediction |
| **INTRODUCTION** | | |
| Background and Rationale | 2 | Explain the scientific background and rationale for the prediction study.  Page 6 – paragraphs 1 and 2 |
| Objectives | 3 | Specify the study objectives and state the specific model(s) that is/are investigated. State if the study concerns the development of the model(s), a validation effort, or both.  Page 7 – paragraph 1 |
| **METHODS** | | |
| Study design and setting | 4* | Specify the key elements of the study design and describe the setting, locations and relevant dates, including periods of recruitment, follow-up and data collection.  Pages 7 and 8 |
| Participants | 5* | Describe eligibility criteria for participants, and sources and methods of selection of participants.  Pages 7 and 8 |
| Variables: definition | 6* | Clearly define all participant characteristics, risk factors and outcomes. Clearly define genetic variants using a widely-used nomenclature system.  Pages 8 – 10. |
| Variables: assessment | 7* | (a) Describe sources of data and details of methods of assessment (measurement) for each variable. (b) Give a detailed description of genotyping and other laboratory methods.  Pages 8 -10. |
| Variables: coding | 8 | (a) Describe how genetic variants were handled in the analyses. (b) Explain how other quantitative variables were handled in the analyses. If applicable, describe which groupings were chosen, and why.  Pages 9 and 10 |
| Analysis: risk model construction | 9 | Specify the procedure and data used for the derivation of the risk model. Specify which candidate variables were initially examined or considered for inclusion in models. Include details of any variable selection procedures and other model-building issues. Specify the horizon of risk prediction (e.g., 5-year risk).  Pages 7 and 8. The risk models had previously been established. An additional model that merged the features of both previously available models was included. |
| Analysis: validation | 10 | Specify the procedure and data used for the validation of the risk model.  Page 9. The risk models were previously established and the current study is a validation of the risk models. |
| Analysis: missing data | 11 | Specify how missing data were handled.  Page 8 and Figure 1. |
| Analysis: statistical methods | 12 | Specify all measures used for the evaluation of the risk model including, but not limited to, measures of model fit and predictive ability.  Page 10 and 11 |
| Analysis: other | 13 | Describe all subgroups, interactions and exploratory analyses that were examined.  Page 11 |
| **RESULTS** | | |
| Participants | 14* | Report the numbers of individuals at each stage of the study. Give reasons for non-participation at each stage. Report the number of participants not genotyped, and reasons why they were not genotyped.  Page 13, Figure 1. The genetic scores were calculated in 3498 of the 4543 children. Missing values for the model was because there was no material for additional genetic analysis. |
| Descriptives: population | 15* | Report demographic and clinical characteristics of the study population, including risk factors used in the risk modeling.  Page 12 and 13 |
| Descriptives: model estimates | 16 | Report unadjusted associations between the variables in the risk model(s) and the outcome. Report adjusted estimates and their precision from the full risk model(s) for each variable.  not applicable |
| Risk distributions | 17* | Report distributions of predicted risks and/or risk scores.  Risk scores: page 12 and Sup Fig 1; Page 14 and Figure 3  Predicted risks: Page 12 and Sup Figure 1; Page 14, 15 and Figure 4 |
| Assessment | 18 | Report measures of model fit and predictive ability, and any other performance measures, if pertinent.  Pages 15 and 16, Figure 5, Supplementary tables 3, 4, 5 |
| Validation | 19 | Report any validation of the risk model(s).  not applicable |
| Other analyses | 20 | Present results of any subgroup, interaction or exploratory analyses, whenever pertinent.  Page 15 and Sup Figure 5 and 6 |
| **DISCUSSION** | | |
| Limitations | 21 | Discuss limitations and assumptions of the study, particularly those concerning study design, selection of participants, measurements and analyses, and discuss their impact on the results of the study.  Page 16 (last paragraph continuing to page 17) |
| Interpretation | 22 | Give an overall interpretation of results considering objectives, limitations, multiplicity of analyses, results from similar studies, and other relevant evidence.  Page 18 |
| Generalizability | 23 | Discuss the generalizability and, if pertinent, the health care relevance of the study results.  Page 17 and 18 |
| **OTHER** | | |
| Supplementary information | 24 | State whether databases for the analyzed data, risk models and/or protocols are or will become publicly available and if so, how they can be accessed.  Data are available from the NIDDK Central Repository  https://www.niddkrepository.org/home/ |
| Funding | 25 | Give the source of funding and the role of the funders for the present study. State whether there are any conflicts of interest.  Pages 18 and 19 |
