# Supplementary material for: Genetic scores to stratify risk of developing multiple islet autoantibodies and type 1 diabetes: A prospective study in children
Source: PLoS Med. 2018 Apr 3;15(4):e1002548. doi: 10.1371/journal.pmed.1002548 (PMC5882115; doi:10.1371/journal.pmed.1002548)
Supplement: S1 Table — (DOC) [file pmed.1002548.s009.doc]

**S1 Table. Weights for single nucleotide polymorphisms used to calculate the genetic scores**

| **SNP** | **Gene, Allele, Genotype** | **Winkler Score Weight** | **Oram Score Weight** | **Merged Score Weight** |
| --- | --- | --- | --- | --- |
| **HLA class II** |  |  |  |  |
| rs17426593  rs2187668  rs7454108 | HLA DR4-DQ8/  DR4-DQ8 | 3.21 | 3.09 | 3.15 |
|  | HLA DR3/  DR4-DQ8 | 4.09 | 3.87 | 3.98 |
| **Other SNPs** |  |  |  |  |
| rs1264813 | HLA A 24 |  | 0.43 | 0.43 |
| rs2395029 | HLA B 5701 |  | 0.92 | 0.92 |
| rs2476601 | PTPN22 | 0.85 | 0.67 | 0.76 |
| rs2816316 | RGS1 | 0.16 |  | 0.16 |
| rs3024505 | IL10 | 0.26 | 0.17 | 0.22 |
| rs1990760 | IFIH1 | 0.16 | 0.15 | 0.16 |
| rs3087243 | CTLA4 | 0.12 | 0.20 | 0.16 |
| rs10517086 | C4orf52 | 0.19 |  | 0.19 |
| rs2069763 | IL2 | −0.01 | 0.11 | 0.11 |
| rs6897932 | IL7RA | 0.19 |  | 0.19 |
| rs3757247 | BACH2 | 0.25 | 0.12 | 0.19 |
| rs9388489 | C6orf173 | 0.12 | 0.16 | 0.14 |
| rs6920220 | TNFAIP3 | 0.15 |  | 0.15 |
| rs1738074 | TAGAP | 0.05 |  | 0.05 |
| rs7804356 | SCAP2 | 0.15 |  | 0.15 |
| rs4948088 | COBL | 0.09 | 0.26 | 0.17 |
| rs7020673 | GLIS3 | 0.23 |  | 0.23 |
| rs12722495 | IL2RA | 0.47 | 0.46 | 0.47 |
| rs947474 | PRKCQ | 0.15 |  | 0.15 |
| rs10509540 | RNLS/C10orf59 | 0.22 | 0.29 | 0.25 |
| rs1004446 | INS | 0.74^b^ | 0.56 | 0.65 |
| rs4763879 | CD69 | 0.06 |  | 0.06 |
| rs2292239 | ERBB3 | 0.41 | 0.30 | 0.36 |
| rs3184504 | SH2B3 | 0.24 |  | 0.24 |
| rs1465788 | ZFP36L1 | 0.12 | 0.15 | 0.13 |
| rs17574546 | RASGRP1 |  | 0.13 | 0.13 |
| rs3825932 | CTSH | −0.20 | 0.15 | 0.15 |
| rs12708716 | CLEC16A | 0.09 | 0.21 | 0.15 |
| rs4788084 | IL27 | 0.25 | 0.15 | 0.20 |
| rs7202877 | CTRB2 | 0.13^b^ | 0.25 | 0.19 |
| rs2290400 | ORMDL3 | 0.25 |  | 0.25 |
| rs7221109 | CCR7 | 0.15 |  | 0.15 |
| rs45450798 | PTPN2 | 0.01 | 0.18 | 0.09 |
| rs763361 | CD226 | 0.10 | 0.15 | 0.12 |
| rs425105 | PRKD2 | 0.26 | 0.15 | 0.21 |
| rs2281808 | SIRPG | 0.05 | 0.10 | 0.07 |
| rs3788013 | UBASH3a | 0.20 | 0.12 | 0.16 |
| rs5753037 | RPS3AP51 | 0.20 | 0.10 | 0.15 |
| rs229541 | IL2B | 0.18 |  | 0.18 |
| rs5979785 | TLR8 | 0.09^b^ |  | 0.09 |
| rs2664170 | GAB3 | 0.14^b^ |  | 0.14 |
| rs917997 | IL18RAP | 0.14^a,b^ |  | 0.14^a^ |

^a^Not included in the genetic score calculations for the TEDDY cohort; ^b^Not included in the Winkler genetic score calculation for the UK cohort and WTTCC patients

SNP, single nucleotide polymorphism; HLA, human leukocyte antigen
