# Supplementary material for: Genetic scores to stratify risk of developing multiple islet autoantibodies and type 1 diabetes: A prospective study in children
Source: PLoS Med. 2018 Apr 3;15(4):e1002548. doi: 10.1371/journal.pmed.1002548 (PMC5882115; doi:10.1371/journal.pmed.1002548)
Supplement: S2 Table — (DOC) [file pmed.1002548.s010.doc]

**S2 Table. Frequencies of risk alleles in TEDDY children with the HLA DR3/DR4-DQ8 or DR4-DQ8/DR4-DQ8 genotypes**

| **SNP** | **Gene, Allele** | **Minor Allele** | **Frequency (%)** | | ***P*-value** |
| --- | --- | --- | --- | --- | --- |
|  |  |  | **Europe** | **USA** |  |
| rs3087243 | CTLA4 | A | 37.28 | 45.28 | 8.30E−11 |
| rs2476601 | PTPN22 | A | 12.11 | 8.8 | 2.42E−05 |
| rs4788084 | IL27 | A | 45.77 | 40.52 | 3.80E−05 |
| rs2069763 | IL2 | A | 40 | 35.5 | 7.61E−05 |
| rs3757247 | BACH2 | A | 41.08 | 45.73 | 0.0003 |
| rs1738074 | TAGAP | A | 40.59 | 44.98 | 0.0004 |
| rs45450798 | PTPN2 | C | 17.17 | 16.12 | 0.0010 |
| rs9388489 | C6orf173 | G | 44.65 | 48.77 | 0.0012 |
| rs2292239 | ERBB3 | A | 33.19 | 29.45 | 0.0018 |
| rs7804356 | SCAP2 | G | 21.64 | 25.11 | 0.0025 |
| rs3184504 | SH2B3 | A | 45.92 | 42.72 | 0.0058 |
| rs2664170 | GAB3 | G | 30.28 | 33.62 | 0.0129 |
| rs5753037 | RPS3AP51 | A | 34.93 | 37.96 | 0.021 |
| rs3788013 | UBASH3a | A | 39.81 | 41.26 | 0.024 |
| rs1990760 | IFIH1 | G | 39.98 | 43.04 | 0.028 |
| rs6897932 | IL7RA | A | 29.3 | 26.83 | 0.037 |
| rs6920220 | TNFAIP3 | A | 21.38 | 19.13 | 0.037 |
| rs1465788 | ZFP36L1 | A | 29.39 | 27.22 | 0.096 |
| rs2816316 | RGS1 | C | 17.65 | 19.09 | 0.1349 |
| rs229541 | IL2B | A | 40.47 | 42.35 | 0.14 |
| rs2395029 | HLA B 5701 | C | 0.96 | 1.46 | 0.15 |
| rs7020673 | GLIS3 | G | 49.15 | 48.35 | 0.16 |
| rs7202877 | CTRB2 | C | 11.74 | 10.45 | 0.18 |
| rs10509540 | RNLS/C10orf59 | G | 27.54 | 25.7 | 0.19 |
| rs7221109 | CCR7 | A | 37.76 | 35.83 | 0.19 |
| rs5979785 | TLR8 | G | 26.55 | 28.87 | 0.21 |
| rs12722495 | IL2RA | G | 8.29 | 9.35 | 0.26 |
| rs2290400 | ORMDL3 | A | 47.87 | 49.74 | 0.29 |
| rs3024505 | IL10 | A | 16.15 | 14.95 | 0.31 |
| rs4763879 | CD69 | A | 38.24 | 36.63 | 0.37 |
| rs3825932 | CTSH | A | 36.13 | 34.89 | 0.37 |
| rs10517086 | C4orf52 | A | 28.71 | 27.93 | 0.38 |
| rs947474 | PRKCQ | G | 18.5 | 18.12 | 0.40 |
| rs4948088 | COBL | A | 4.51 | 5.15 | 0.45 |
| rs17574546 | RASGRP1 | C | 20.23 | 19.34 | 0.60 |
| rs1264813 | HLA A 24 | A | 9.51 | 8.98 | 0.63 |
| rs12708716 | CLEC16A | G | 34.85 | 33.96 | 0.65 |
| rs425105 | PRKD2 | G | 16.24 | 15.63 | 0.74 |
| rs1004446 | INS | A | 37.51 | 37.15 | 0.77 |
| rs2281808 | SIRPG | A | 33.26 | 33.95 | 0.83 |
| rs763361 | CD226 | A | 48.45 | 48.12 | 0.86 |
