# Supplementary material for: Genetic scores to stratify risk of developing multiple islet autoantibodies and type 1 diabetes: A prospective study in children
Source: PLoS Med. 2018 Apr 3;15(4):e1002548. doi: 10.1371/journal.pmed.1002548 (PMC5882115; doi:10.1371/journal.pmed.1002548)
Supplement: S3 Table — (DOC) [file pmed.1002548.s011.doc]

**S3 Table. Risk of developing one or more islet autoantibodies by age 6 years and the proportion of cases positive for any islet autoantibodies (sensitivity) in TEDDY children with the HLA DR3/DR4-DQ8 or DR4-DQ8/DR4-DQ8 genotypes stratified by their merged TEDDY Score, with corresponding 95% confidence intervals (CIs). The risk and sensitivity are shown for each increment in the genetic score by the 5th percentile of scores in the TEDDY children with the HLA DR3/DR4-DQ8 or DR4-DQ8/DR4-DQ8 genotypes ranging from >12.1 (lower 5th percentile of children) to >15.4 (upper 5th percentile of children).**

| **Risk score cut-off** | **Cumulative risk (95% CI)** | **Sensitivity (95% CI)** |
| --- | --- | --- |
| 12.1 | 9.5 (8.4, 10.6) % | 97.8 (95.4, 99.0) % |
| 12.4 | 9.9 (8.7, 11.0) % | 96.4 (93.5, 98.0) % |
| 12.7 | 10.0 (8.9, 11.2) % | 92.4 (88.7, 95.0) % |
| 12.9 | 10.2 (9.0, 11.4) % | 88.4 (84.1, 91.7) % |
| 13.1 | 10.6 (9.3, 11.8) % | 85.6 (80.9, 89.2) % |
| 13.2 | 10.8 (9.5, 12.2) % | 82.3 (77.4, 86.4) % |
| 13.4 | 11.2 (9.8, 12.6) % | 79.1 (73.9, 83.4) % |
| 13.5 | 11.6 (10.1, 13.1) % | 75.8 (70.4, 80.5) % |
| 13.6 | 12.2 (10.6, 13.7) % | 72.9 (67.4, 77.8) % |
| 13.8 | 12.5 (10.8, 14.2) % | 68.2 (62.5, 73.4) % |
| 13.9 | 12.8 (11.0, 14.6) % | 63.2 (57.4, 68.6) % |
| 14.0 | 13.7 (11.7, 15.6) % | 59.2 (53.3, 64.8) % |
| 14.2 | 14.2 (12.0, 16.3) % | 53.8 (47.9, 59.6) % |
| 14.3 | 14.8 (12.4, 17.1) % | 48.4 (42.6, 54.2) % |
| 14.4 | 16.0 (13.3, 18.6) % | 43.7 (38.0, 49.6) % |
| 14.6 | 16.2 (13.2, 19.1) % | 35.0 (29.6, 40.8) % |
| 14.8 | 17.5 (13.8, 21.0) % | 27.8 (22.9, 33.3) % |
| 15.1 | 19.3 (14.6, 23.7) % | 20.6 (16.2, 25.7) % |
| 15.4 | 20.4 (13.7, 26.6) % | 11.2 (8.0, 15.4) % |
