# Supplementary material for: Genetic scores to stratify risk of developing multiple islet autoantibodies and type 1 diabetes: A prospective study in children
Source: PLoS Med. 2018 Apr 3;15(4):e1002548. doi: 10.1371/journal.pmed.1002548 (PMC5882115; doi:10.1371/journal.pmed.1002548)
Supplement: S4 Table — (DOC) [file pmed.1002548.s012.doc]

**S4 Table. Risk of developing multiple islet autoantibodies by age 6 years and the proportion of cases positive for multiple islet autoantibodies (sensitivity) in TEDDY children with the HLA DR3/DR4-DQ8 or DR4-DQ8/DR4-DQ8 genotypes stratified by their merged TEDDY Score, with corresponding 95% confidence intervals (CIs). The risk and sensitivity are shown for each increment in the genetic score by the 5th percentile of scores in the TEDDY children with the HLA DR3/DR4-DQ8 or DR4-DQ8/DR4-DQ8 genotypes ranging from >12.1 (lower 5th percentile of children) to >15.4 (upper 5th percentile of children).**

| **Risk score cut-off** | **Cumulative risk (95% CI)** | **Sensitivity (95% CI)** |
| --- | --- | --- |
| 12.1 | 6.2 (5.3, 7.0) % | 100 (97.8, 100) % |
| 12.4 | 6.4 (5.5, 7.4) % | 98.8 (95.9, 99.7) % |
| 12.7 | 6.6 (5.6, 7.6) % | 96.0 (91.9, 98) % |
| 12.9 | 6.8 (5.7, 7.8) % | 92.5 (87.6, 95.6) % |
| 13.1 | 7.0 (5.9, 8.1) % | 89.6 (84.2, 93.3) % |
| 13.2 | 7.3 (6.2, 8.4) % | 87.3 (81.5, 91.5) % |
| 13.4 | 7.5 (6.3, 8.7) % | 83.2 (77.0, 88.1) % |
| 13.5 | 7.7 (6.5, 9.0) % | 79.8 (73.2, 85.1) % |
| 13.6 | 8.1 (6.7, 9.4) % | 76.3 (69.4, 82.0) % |
| 13.8 | 8.2 (6.8, 9.6) % | 71.1 (63.9, 77.3) % |
| 13.9 | 8.6 (7.1, 10.1) % | 67.1 (59.7, 73.6) % |
| 14.0 | 9.1 (7.5, 10.8) % | 62.4 (55.0, 69.3) % |
| 14.2 | 10.1 (8.2, 11.9) % | 60.1 (52.7, 67.1) % |
| 14.3 | 10.2 (8.2, 12.2) % | 52.6 (45.2, 59.9) % |
| 14.4 | 11.0 (8.7, 13.3) % | 47.4 (40.1, 54.8) % |
| 14.6 | 11.9 (9.2, 14.5) % | 40.5 (33.4, 47.9) % |
| 14.8 | 12.0 (8.9, 15.1) % | 30.1 (23.7, 37.3) % |
| 15.1 | 13.2 (9.2, 17.1) % | 22.0 (16.4, 28.7) % |
| 15.4 | 12.2 (6.7, 17.4) % | 10.4 (6.7, 15.8) % |
