# Supplementary material for: Genetic scores to stratify risk of developing multiple islet autoantibodies and type 1 diabetes: A prospective study in children
Source: PLoS Med. 2018 Apr 3;15(4):e1002548. doi: 10.1371/journal.pmed.1002548 (PMC5882115; doi:10.1371/journal.pmed.1002548)
Supplement: S5 Table — (DOC) [file pmed.1002548.s013.doc]

**S5 Table. Risk of developing type 1 diabetes by age 10 years and the proportion of cases positive for type 1 diabetes (sensitivity) in TEDDY children with the HLA DR3/DR4-DQ8 or DR4-DQ8/DR4-DQ8 genotypes stratified by their merged TEDDY Score, with corresponding 95% confidence intervals (CIs). The risk and sensitivity are shown for each increment in the genetic score by the 5th percentile of scores in the TEDDY children with the HLA DR3/DR4-DQ8 or DR4-DQ8/DR4-DQ8 genotypes ranging from >12.1 (lower 5th percentile of children) to >15.4 (upper 5th percentile of children).**

| **Risk score cut-off** | **Cumulative risk (95% CI)** | **Sensitivity (95% CI)** |
| --- | --- | --- |
| 12.1 | 4.2 (3.3, 5.1) % | 100 (96.5, 100) % |
| 12.4 | 4.4 (3.4, 5.4) % | 100 (96.5, 100) % |
| 12.7 | 4.6 (3.6, 5.6) % | 99.1 (94.9, 100) % |
| 12.9 | 4.6 (3.6, 5.6) % | 96.3 (90.8, 98.5) % |
| 13.1 | 4.8 (3.7, 5.8) % | 93.5 (87.1, 96.8) % |
| 13.2 | 5.1 (4.0, 6.2) % | 92.5 (85.9, 96.2) % |
| 13.4 | 5.1 (4.0, 6.2) % | 87.9 (80.3, 92.8) % |
| 13.5 | 5.3 (4.1, 6.4) % | 84.1 (76.0, 89.8) % |
| 13.6 | 5.5 (4.3, 6.8) % | 81.3 (72.9, 87.6) % |
| 13.8 | 5.6 (4.3, 6.9) % | 74.8 (65.8, 82.0) % |
| 13.9 | 5.9 (4.5, 7.4) % | 71.0 (61.8, 78.8) % |
| 14.0 | 6.2 (4.6, 7.7) % | 65.4 (56.0, 73.8) % |
| 14.2 | 7.0 (5.2, 8.7) % | 64.5 (55.1, 72.9) % |
| 14.3 | 7.0 (5.0, 8.9) % | 55.1 (45.7, 64.2) % |
| 14.4 | 7.6 (5.3, 9.9) % | 48.6 (39.3, 58.0) % |
| 14.6 | 7.8 (5.4, 10.1) % | 42.1 (33.1, 51.5) % |
| 14.8 | 8.3 (5.4, 11.0) % | 33.6 (25.4, 43.0) % |
| 15.1 | 9.5 (5.7, 13.1) % | 25.2 (18.0, 34.2) % |
| 15.4 | 9.3 (3.5, 14.8) % | 12.1 (7.2, 19.7) % |
